# Breastfeeding in Italy. How “the first 1,000 days” discourse molecularises social expectations of intensive mothering

**DOI:** 10.3389/fsoc.2025.1671596

**Published:** 2025-11-19

**Authors:** Daniela Bandelli

**Affiliations:** Department of Political and Social Studies, University of Salerno, Fisciano, Italy

**Keywords:** breastfeeding, sociology of health, intensive parenting, motherhood, child’s health, public health campaigns, epigenetics

## Abstract

**Introduction:**

Breastfeeding is one of the core pillars of the so-called “First Thousand Days” (FTD) discourse. By mobilising neuroscience, the Developmental Origins of Health and Disease (DOHaD), and epigenetics, this contemporary narrative establishes a causal link between various pre-natal and early-life lifestyle factors and health across the lifespan. By framing parental choices as social determinants of children’s health, it aligns with broader contemporary parenting trends, such as scientific motherhood and intensive parenting, the expectation that parents, particularly mothers, devote significant time and energy to raising their children according to the latest scientific advice.

**Methods:**

A qualitative analysis of health information guides and policy papers circulating in Italy over the last 6 years was conducted following the principles of Critical Discourse Analysis (CDA). The aim was to explore how the promotion of breastfeeding within the FTD framework normalises biomedical imaginaries of childrearing and increases social pressure on mothers.

**Results:**

The analysed texts emphasise individual behavioral prescriptions for mothers, focusing on nutrition, bodily techniques, and information gathering, while largely overlooking structural barriers such as inadequate parental leave or poor work-life balance. Biomedical and epigenetic narratives portray the mother as a vector for the child’s gene expression, development, and health. She is positioned as dependent on expert guidance, while embodied maternal knowledge is marginalised.

**Discussion:**

This discourse blends social and biological determinism, reinforcing intensive mothering ideals rooted in healthism, and underestimating the structural constraints that hinder full adherence to these expectations. In the Italian context, characterized by weak parental support policies and limited implementation of breastfeeding promotion, this narrative may contribute to a perception of motherhood as anomic, where the ideal of raising healthy children is promoted without providing the necessary means to achieve it.

## 1 Introduction

Breastfeeding is a biopsychosocial practice (Asimaki et al., 2022) that demands a significant amount of time (Smith and Forrester, 2013), biological effort, emotional engagement, concentration, and, for some women, hardship as well as bodily discomfort and pain (Caes et al., 2021). Historically, breastfeeding has been considered the most appropriate way to provide nutrition for infants, guided by both traditional practices and scientific knowledge. In contemporary society, however, it is widely recognized as the gold standard of care, as evidenced by a biomedical discourse that highlights the micro-properties of breast milk (such as hormones and vitamins), its nutritional and immune benefits, and its impact on infant health and cognitive development (Belfort, 2017; Swanson, 2009; Torres, 2014). In other words, scientific and medical knowledge has played a significant role in shaping our understanding of breastfeeding as a “natural extension of childbirth, seen as a crucial part of the nurturing process for children” (Augusto et al., 2023, p. 1).

Breastfeeding, along with infant nutrition, is one of the core pillars of the so-called First Thousand Days (FTD) discourse, which, in Foucauldian terms, can be defined as a “device” (or *dispositif*, see Raffnsøe et al., 2014) of cultural change in parenting: a powerful set of assumptions, expectations, and explanations that reshapes present and past beliefs about what are supposed to be the expected concerns, behaviors, goals, daily activities, sources of knowledge, and self-perceptions of parents. A *dispositif* points to new ways of thinking and orients people’s opinions and actions; in other words, it governs mainstream social and cultural practices, providing “systematic ways of making sense of the world” (Baxter, 2003, p. 7). By mobilising neuroscience, the Developmental Origins of Health and Disease (DOHaD), and epigenetics, the FTD discourse establishes a causal, mechanistic link between various preconception, prenatal, and early infancy conditions and long-term health outcomes (Pentecost and Ross, 2019).

Increasingly validated by the medical community, policy-making, and public awareness over the last decade, the FTD narrative has been disseminated in contemporary parenting culture through institutional guidelines, medical advice, scientific publications, awareness initiatives, prenatal classes, and parenting workshops. It encourages parents to pay attention to various aspects of family life and parental care - such as nutrition, drug consumption, lifestyle, screen exposure, medical checkups, pedagogical approaches, exposure to art, housing, pollution, etc. - in order to influence fetal programming, metabolic adaptation, brain rewiring, and the activation of the “right” genes, as well as the robustness of the child’s immune system (Aguayo and Britto, 2024). Therefore, the FTD discourse is one of the recent health-risk narratives contributing to the culture of intensive parenting and to the phenomenon of scientific motherhood: the social expectation that parents, particularly mothers, dedicate “tremendous amounts of time, energy, and money” to raising their children (Hays, 1996), and do what is best for their child’s well-being and future prospects by following the latest scientific advice (Apple, 2006).

This article focuses on breastfeeding recommendations within the FTD discourse as it is applied in Italy, a country with one of the lowest fertility rates in Europe and suboptimal breastfeeding incidences, a hyper-medicalised birth process (one of the contributing factors to breastfeeding complications), widespread reliance on medical advice in childrearing, and a lack of postpartum home services (Bimbi and Toffanin, 2017; Castagnaro and Prati, 2015; DeMaria et al., 2020; Spina, 2023; ISS Allattamento, 2025).

In light of the fact that breastfeeding campaigns - particularly when conveyed through a biomedical lexicon - are likely to reinforce social pressure on “normative mothers,” who are “increasingly charged with taking care of the molecular level of their children’s future” (Blum and Fenton, 2016, p. 100), a qualitative analysis was conducted on a sample of health information guides and policy documents circulating in Italy. Drawing on the principles of Critical Discourse Analysis (CDA), this study explores the extent to which the promotion of breastfeeding within the FTD narrative contributes to the hyper-responsibilisation of mothers for their children’s health outcomes, and whether it takes into account the social contexts and material constraints that may limit their capacity to act accordingly. The analysis is grounded in the assumption that the FTD discourse—by promoting a biomedical imaginary of breastfeeding and the mother–child relationship—intensifies social expectations placed on mothers and deepens their reliance on medical expertise in everyday childrearing practices.

The article unfolds through the following sections. The first section reviews existing literature in the sociology of health, Science and Technology Studies (STS), and interdisciplinary research on breastfeeding, focusing on how the dissemination of scientific knowledge in epigenetics and neuroscience has shaped the definition of the mother’s role in her children’s health. The second section outlines the methodology and, in defining the spirit of CDA, also clarifies the study’s objectives. The third section is dedicated to interpreting the results of the analysis and is organized into three sub-themes: the representation of breastmilk as unique and beneficial; interventions to support breastfeeding, with an emphasis on individual behavioral prescriptions; and the biomedical imagery shaping the subject positions of the mother. Finally, the conclusion discusses the social implications of this discourse, including a form of anomic mothering arising from the tension between the promoted ideal of raising healthy children and a social context characterized by inadequate support and insufficient resources to implement behavioral prescriptions.

### 2 The (milking) mother’s responsibility for the child’s health across epigenetics and neurosciences

The FTD discourse asserts that from the pre-pregnancy period (Waggoner, 2017) - or at least from conception - to the second year of life, parents should control several factors, such as trauma and stress, intellectual stimulation, exposure to pollutants, and nutrient intake, that may influence gene expression, and thus the child’s development and long-term health. This is because during this time, the human body, and especially the brain, is considered highly malleable, and this malleability is framed as a valuable opportunity to foster resilience in future adults and reduce vulnerability to chronic diseases (Harris and McDade, 2018). The FTD discourse has been enriched by a growing interest in children’s health and development within public policy, intersecting with the pervasive biomedicalisation of all aspects of life and care, the culture of health risk, and the dominant model of intensive parenting and scientific motherhood (Apple, 2006; Hays, 1996; Lupton, 2011).

Neuroscience and epigenetics - the study of how environmental factors may influence the phenotype by steering gene modification - are two related fields that provide a scientific basis for some contemporary beliefs about childrearing and breastfeeding. Notably, these fields contribute to the idea that parenting is both the main cause and the solution for various developmental conditions in children (Lowe et al., 2015). The popularity of molecular epigenetics has lent support to earlier epidemiological hypotheses suggesting that several illnesses may originate in the fetus’s or infant’s response to the mother’s biophysical activities (Chiapperino and Panese, 2018). At the same time, neuroscience has contributed to a shift in the public image of the brain from a computational machine to a malleable organ that depends on social relationships and can be impaired by trauma and emotional deprivation (Auriemma, 2023; Meloni and Testa, 2014). Together, these scientific research streams contribute to the notion that the prenatal period and the first years of life are critical windows for programming genetic and developmental pathways in areas such as metabolism and brain development.

Scholars observe that one side effect of narratives that “molecularise” the body is that health risk mitigation becomes a matter of personal responsibility for parents (Lappé and Hein, 2022; Mansfield, 2012), while structural factors beyond parental capacity - such as social stratification, public policy, and health literacy - are often overlooked (Brady et al., 2015). The molecularisation process was already at work during the genomic era, when it was believed that individuals could not intervene in their “unique” biological destiny, and it continues to persist after the epigenetic turn, an emerging scientific paradigm that has helped bridge social dynamics and biological processes (Meloni and Müller, 2018). This paradigm shift has contributed to a transition from genetic determinism to a biopsychosocial model of health and the body, in which individuals and environments play active roles in altering gene function (Meloni and Müller, 2018; Meloni and Testa, 2014). Within this new framework, the risk of poor health outcomes shifts from the body’s internal nature to the individual’s capacity to manage exposure to external risks and benefits, which in turn impacts gene behavior.

As feminist studies and the sociology of health have extensively pointed out (see Scavarda, 2024; Sommerfeld, 1989, among others), mothers have always been held responsible for procreative success and their children’s health, and these social expectations predate epigenetics and neuroscience by a long time. In the FTD discourse, however, these fields constitute the new argumentative basis for consolidating the link between maternal behaviors - during pregnancy and the child’s infancy - and a child’s life: the female reproductive body is construed through a molecular gaze as an epigenetic carrier (Richardson, 2021), and the mother is held responsible for managing environmental conditions that can be either adverse or favourable to the child’s health and development. Thus, women are expected to engage actively in self-surveillance of their bodies and lifestyles in adherence to medical expert advice (Lappé and Hein, 2022). It is noteworthy that through the epigenetic paradigm, the temporal reach of maternal behavioral influence is extended to the period before conception and across the gene lineages of future generations (Mansfield, 2017; Waggoner, 2017). In this vein, neuroscience and epigenetics have provided a “layer of biological undergirding or determinacy” to “nurture-based intensive mothering norms” (Blum and Fenton, 2016, p. 108) and have solidified the traditional perception of the maternal body as potentially dangerous to the child and thus a target of interventions (Martin, 1987; Richardson, 2021). During the postnatal period, these arguments encourage mothers to engage in on-demand and extended breastfeeding or, as a second option, to feed their children with expressed milk.

It is noteworthy that human milk has always been the primary source of nutrition for newborns, and until recently, the completion of human reproduction depended on breastfeeding (either from the child’s mother or another woman). In exceptional circumstances, such as the death of the mother or the unavailability of a wet nurse, it was customary to use cow’s milk or plant-based preparations, which, regrettably, often resulted in health complications and even the death of the child (Cohen, 2017). Following World War II, significant progress in technology and science paved the way for the development of formulas derived from non-human sources, believed to provide adequate nourishment for newborns without the need for maternal participation. For the first time, there was collective enthusiasm for a human artefact which, according to the medical and commercial discourse of the time, appeared to allow the reproductive process to be completed with a performance seemingly equivalent to or better than traditional nourishment (Foss, 2010). Since the 1990s, there has been increasing focus on preserving, processing, and distributing human matter, as well as studying its qualities and possible therapeutic applications (e.g., milk banks, nutritional and pharmaceutical products) (Swanson, 2009). Influenced by “lactivism”- a social movement seeking social recognition and appropriate spaces for breastfeeding (Faircloth, 2013) - and public health campaigns informing new mothers about the differences between human milk and powdered milk, human milk has once again been recommended as the optimal source of nutrition.

One notable development that followed has been the promotion of the “breast is best” message, alongside the increasing use of breast pumps, which allow mothers’ own milk to be fed to infants by fathers or other caregivers in the mother’s absence (Crossland et al., 2016). While this second option offers a strategy to achieve the ideal goal of providing children with optimal nutrition without requiring mothers to forgo their professional lives, it also involves additional work and fatigue for both parents. Tasks such as freezing, heating, measuring portions, and cleaning equipment add to their daily workload (Johnson, 2019). Moreover, women may experience a sense of alienation when feeding time is replaced by milk expression, which lacks the emotional connection and intimacy of direct breastfeeding (Hentges and Pilot, 2021).

Both nutritional modalities are supported by a molecular approach focused on biological processes (Niewöhner, 2011; Rose, 2007). The second modality emphasizes the organoleptic qualities of the fluid, while the first describes the biological processes of the mother’s and child’s bodies. For example, the idea that “breast is best” is explained by describing how the encounter of saliva with milk triggers a chemical reaction producing a substance useful in fighting salmonella; it also explains how only through sucking is a hormone produced that signals the baby to feel full, and how the baby sends signals to the mother to produce, at that precise moment, the substances it needs in the milk (Modi, 2024).

## 3 Methodology: applying critical discourse analysis in public health

CDA combines linguistic and thematic text analysis with Critical Theory, notably adopting an emancipatory approach to sociological analysis (Fairclough, 1995). When applied in public health, CDA can serve as a tool to question the biomedical approach used in awareness campaigns, aiming to better align social policies with people’s lived experiences (Naidu et al., 2023). By suggesting that the assumptions underpinning public health campaigns may be open to debate, CDA highlights how science is sometimes mobilized in public policies to evoke emotional responses (Segal, 2009). Rather than determining the truth or falsity of the scientific knowledge mobilized, the goal of CDA is to illuminate the relationships between discourse, practice, and knowledge (Janks, 1997). In this sense, texts are analysed not to criticize them per se, but to contribute to social critique by uncovering the assumptions embedded in social practices. In this regard, the FTD discourse is understood as a form of knowledge that, although neither universally accepted as true nor empirically verifiable (Fairclough, 1995), has nonetheless become deeply entrenched as the legitimate style of contemporary parenting. Additionally, CDA enables examination of how different subjects are constructed within a given narrative, legitimizing certain forms of knowledge and practices while delegitimizing others (Arribas-Ayllon and Walkerdine, 2008). In this vein, it is particularly relevant to investigate how mothers, and parents more broadly, are positioned within the FTD discourse on breastfeeding. In this research, CDA was guided by the overarching aim of assessing the extent to which the framing of breastfeeding within the contemporary FTD narrative contributes to legitimizing references to biomedical or molecular imagery in child-rearing, thereby reinforcing social pressure on mothers and fathers to assume individual responsibility for their children’s health and to follow expert advice.

The texts were identified through a simple keyword search on Google (“i primi mille giorni”) and only texts that were fully and explicitly dedicated to the contemporary FTD discourse were selected. The sample includes texts published primarily by medical authorities and professional medical organizations, alongside two publications by civil society organizations. Only texts with a nationwide scope were selected for the analysis. The intended audience includes the general public, parents, and policymakers. While not exhaustive, the sample is intended to serve as an illustrative representation of the contemporary discourse surrounding FTD as it has emerged in Italy over the past 5–6 years.

– 2019 Italian Ministry of Health strategy targeting health professionals, parents, and policymakers. Title: *Early Investment in Health: Actions and Strategies in the First 1,000 Days*.^1^
– Pamphlet published in 2020 by Bambino Gesù Hospital, one of Italy’s main paediatric hospitals. Title: *Learning About Health: The First 1,000 Days*.^2^
– Pamphlet published in 2021 aimed at raising fathers’ awareness of their role in breastfeeding. Result of the EC-funded PARENT project realized by several partners, including the Istituto Superiore di Sanità and fathers’ collectives. Title: *Parent*.^3^
– 2022 Report on Childhood in Italy by Save the Children, including a chapter dedicated to the FTD. Title: *Vulnerable Childhood*.^4^
– Website created in 2023 as part of a project initiated in 2017 by the National Center for Disease Prevention and Control (CCM), financially supported by the Ministry of Health and the Friuli Venezia Giulia Region, in partnership with the regions of Piedmont, Tuscany, Lazio, and Sicily. Title: *The First 1,000 Days and the Environment*.^5^
– Policy brief published in 2024 by Centro per la Salute dei Bambini Onlus, in partnership with several civil society organizations engaged in childbirth, midwives, paediatricians associations, and the Istituto Superiore di Sanità. Title: *On Tiptoe. Individual Meetings and Home Visits from Pregnancy to the First Months of Life*.^6^
– -Health booklet for parents, published in 2025 by the national newspaper *Il Corriere della Sera* in consultation with the Italian Society of Paediatrics and the Lombardy Health Foundation. Title: *The First 1,000 Days Decisive for Health*.^7^

For the purpose of the study, relevant paragraphs from each text were selected and analysed using a circular approach. This method involved iterative transitions between close readings of the texts, guided by the research questions. The researcher also engaged critically with the texts, reflecting on her own assumptions regarding intensive parenting, breastfeeding normativity, and medicalization of life. This approach, which combines analytical rigor, theoretical grounding, and reflexivity, enhances the overall credibility of the research findings (Wodak and Meyer, 2009). By integrating both inductive and deductive strategies for qualitative coding, the following three overarching questions were progressively refined into sub-questions that informed the coding categories (Naeem et al., 2023). The questions and sub-questions are as follows:

1. Breastfeeding representation and effects: What are the key qualifications for breastfeeding (e.g., action, nutrition, care, choice, etc.)? What spillovers or benefits are related to lactation?
2. Actions to support breastfeeding: Which behavioral prescriptions are recommended to parents? To what extent do these take into account the social conditions and material constraints mother may face? What other social or public health interventions are proposed to promote breastfeeding?

The analysis was carried out manually, which allowed for complete immersion in the text, capturing its nuanced meanings and leveraging critical thinking (Braun and Clarke, 2006). For each macro question, a worksheet was completed, documenting relevant phrases or words. Each document was then reread to answer the following cross-cutting questions:

1. The biomedical imagery: How is the molecular gaze mobilized to establish the relationship between breastfeeding and health outcomes? What type of relationship is it (causal, multifactorial, etc.)? Are epigenetics and neuroscience mobilized to justify behavioral recommendations?
2. Subject positions - How are mothers positioned or represented (e.g., in terms of their capabilities, responsibilities, choice, etc.)? What roles, if any, are assigned to fathers in breastfeeding? How is the mother positioned in relation to experts and health professionals: as a passive recipient of expert knowledge, or as an active subject within the health discourse?

The analytical process was guided by the overarching research aim, the macro and cross-cutting questions, and the relevant literature, all of which were revisited in a circular manner throughout multiple stages of the textual analysis.

## 4 Breastfeeding in “the first 1,000 days” discourse in Italy

### 4.1 Benefits and uniqueness of breastfeeding

The analysed texts generally agree on the importance of exclusively breastfeeding a child for the first 6 months of life, and of continuing breastfeeding up to 2 years of age. The WHO’s *Global Strategy for Infant and Young Child Feeding* and *Nurturing Care* serves as the main institutional source legitimizing this recommendation. Breastfeeding is said to have a positive impact on a child’s future development and health, as well as on that of future generations. Among the cited benefits are proper development of organs and tissues, including the brain. The brain is described as plastic, and breastfeeding is linked to cognitive and emotional development. It is also associated with the prevention of various health issues, including asthma, diarrhea, otitis, metabolic diseases, diabetes, obesity, leukaemia, and chronic illnesses in general. Breastfeeding is further promoted as a way to strengthen the immune and sensory systems, protecting children from external risks throughout their lives. Additionally, it is recommended for promoting maternal physical and mental health, being associated with protection against certain cancers and osteoporosis, reduced risk of postpartum haemorrhage, enhanced self-confidence in the mother–child relationship, and support in returning to pre-pregnancy weight.

Breast milk and lactation are associated with uniqueness, essentiality, and extraordinary perfection. In other words, they are linked to the body’s unique potential: terms such as “an essential nutritional and relational contribution,” “an extraordinary synthesis of biological and psychological contributions,” and “a complete” or “inimitable food” are frequently used in the analysed texts. The uniqueness of breastfeeding is causally and deterministically associated with health outcomes, as discussed in the previous paragraph. Additionally, breastfeeding is encouraged as it facilitates family organization: it is described as “practical,” “free,” and “always available,” implicitly contrasting it with formula and expressed milk. Breastfeeding is also presented as “a sustainable choice” because “it requires no production, packaging, or transportation, reducing environmental impact.” It is even characterized as “a gesture of love for the planet”.

This characterization of breastfeeding as an “inimitable” and “extraordinary” biological process, directly linked to the aforementioned benefits, contributes to its normative status. Although breastfeeding is formally acknowledged as a “choice,” this framing appears largely rhetorical, implicitly suggesting that a mother who does not breastfeed is depriving her child of these benefits. According to the Ministry of Health guidelines, “the lack of breast milk or donor milk feeding” is identified as “a risk factor” for “neonatal and infant morbidity, due to altered neurobiological and epigenetic imprinting, also mediated by the microbiome, which regulates immune and metabolic responses, adipogenesis, brain development, and cognitive functions, with long-term effects.” However, the same document states that “mothers who, for medical reasons or by choice, do not breastfeed must be reassured and supported in adequately and effectively feeding their babies, while still building an intense and engaging relationship; this includes fostering a deep bond with the baby by offering comfort, physical contact, calmness, and consistent guidance, with an emotional contribution that is more than sufficient. In these specific cases, specially adapted infant formulas should be used, excluding the use of cow’s milk or milk from other sources”.

Some passages in the analysed texts acknowledge that breastfeeding can cause painful physical issues, such as rhagades, as well as intense physical and mental fatigue, and difficulties in balancing work or taking prescribed medications. These acknowledgments offer a modest challenge to the overall idealization of breastfeeding promoted within the FTD discourse.

Breastfeeding is also described as an “experience” for the mother and as a “physical and emotional relationship” between mother and child that is both unique and essential. Although the relational dimension is not central to the broader discourse, when it is mentioned, it is typically framed in utilitarian terms, valued primarily for its functional role in supporting the child’s growth, well-being, and health, and even in preventing the risk of child maltreatment. An empathic relationship is encouraged, based on the belief that “this can foster a sense of curiosity,” which may contribute to the development of healthier eating habits during childhood.

### 4.2 Actions to support breastfeeding

The analysed Ministry of Health guidelines clearly state that breastfeeding support is a cornerstone of health promotion during the FTD. Among the various recommended changes to maximize health opportunities within this critical period, the greatest emphasis is placed on individual behaviors. Behavioral prescriptions directed at mothers focus on three key dimensions: nutrition, body techniques, and information.

Regarding the first dimension, mothers are advised to follow a diet high in fiber, low in sugar, and balanced in protein, including weekly servings of fish. This regimen should be complemented by adequate hydration and vitamin supplementation, with an emphasis on organic, home-cooked foods. Vegan and vegetarian mothers are recommended to “consume legumes, nuts, and tubers in combination with cereals to restore the profile of essential amino acids”.

“The composition of milk in terms of quality fats—particularly Polyunsaturated Fatty Acids and Docosahexaenoic Acid—is also linked to the mother’s diet: this is why breastfeeding women should continue to consume 2 to 4 servings of oily fish per week, to ensure an adequate intake of valuable polyunsaturated fatty acids, which are essential for brain and visual development”.

Body techniques, which Mauss (1934) defines as the ways in which human beings, in different societies, make use of their bodies, include practical advice on on-demand feeding and body positions such as sitting with a pillow behind the back.

“The semi-reclined breastfeeding position” is recommended as “a true ally for those first feeds and for a calm, natural breastfeeding experience.” This recommendation is supported by references to the natural functioning of both the baby’s and the mother’s bodies, such as the activation of “innate reflexes.” The position aligns with early feeding patterns and “sleeping behaviors” of newborns, making it easier to feed frequently, even during light sleep. Additionally, “skin-to-skin contact” enhances mutual bonding and confidence, while the posture “lowers the risk of nipple pain by promoting a more effective latch”.

Also, techniques to replace -if necessary - breastfeeding with bottle feeding of expressed milk is described in details (such as the use of breast pump, refrigerator storage, and milk transport when returning to work).

“Start expressing milk a few days before returning to work to build up a small reserve. For the first sessions, choose calm moments, such as after the baby has fallen asleep”.

“Freshly expressed milk can be stored in the refrigerator, in the coldest part, for up to 5 days, or frozen for up to 6 months”.

Breastfeeding success or failure is influenced by factors that fall beyond the mother’s capacity and needs structural interventions. Among these are the childbirth method and the assistance received such as the preference for vaginal delivery, rooming-in, and skin-to-skin contact practices provided by Baby-Friendly Hospitals that apply the Mother-Friendly Care protocol. It is also mentioned that there is a need to protect mothers from the aggressive marketing of breast milk substitutes and to ensure that they are provided already during pregnancy with adequate information regarding breastfeeding and/or feeding their infants with human milk, and that they are supported in doing so even when infants are in intensive care. Additionally, there is a growing emphasis on integrating prenatal and postpartum services with territorial services through home visits to support families immediately after delivery during the postpartum period.

It is noteworthy that the texts offer no structural recommendations regarding parental leave, despite some acknowledging that inadequate work-life balance policies pose significant barriers to breastfeeding. This omission may stem in part from the texts’ predominant focus on health from a biomedical perspective. However, it also highlights how the concept of health remains narrowly defined, with limited integration of sociological insights and social policy considerations.

### 4.3 The biomedical imagery and the subject positions

The analysis supports the view, as highlighted in the cited literature, that a molecular gaze and an epigenetic paradigm underpin the breastfeeding recommendations within the FTD discourse. More specifically, not only is milk portrayed as a beneficial substance produced by the female body, but mothering itself - and the care relationship with the child - is also construed as a physiological process. Breast milk is described both as a “fluid” and as a “perfect biological system,” with its micro-components (such as lactoferrin, chitinase, and oligosaccharides) explained in detail to mothers. These substances are said to interact with the infant’s body through complex stimulus–response mechanisms, allowing mothers, through breastfeeding, to activate and preserve the child’s “innate biological competencies.”

The languages of biology and epigenetics are also used to describe the relationship between this fluid and the child’s health, as well as the role of mothers in this mechanism. For instance, it is suggested that “milk trains the immune system” by transferring the “mother’s defensive arsenal,” capable of blocking pathogens. Lactation is also presented as a key factor in shaping the maternal–fetal microbiota, which in turn may influence immune system programming and long-term risk responses. This emphasis on microbiome care represents one of the most recent examples of the hyper-responsibilisation of mothers through the scrutiny of their body’s micro-components (Pala and Kenny, 2025).

It is worth noting that the relationship between maternal behaviors, the quality of lactation, and child health outcomes is depicted as a complex, multifactorial system in which causal links are difficult to control. Lactation is embedded in an intricate ecology of biological processes, individual actions, and environmental conditions.

“The first 1000 days are marked by the extreme complexity and rapid pace of developmental processes, during which an adequate intake of energy and essential nutrients is crucial to support growth, the development of the nervous system, the immune system, and hormonal balance. This period also influences the composition of the gut microbiota, the bacterial flora that regulates the so-called gut-brain axis”.

In this particular ecology, the mother is not portrayed as an autonomous decision-maker equipped with the necessary skills and knowledge to raise her child (Apple, 2006). On the contrary, she is expected to consult a wide range of experts during pregnancy and the postpartum period in order to receive accurate information and resolve doubts, ranging from breastfeeding techniques and timing, to breastfeeding while on medication, the introduction of solid foods after 6 months, and the assessment of the baby’s growth. These experts include paediatricians, midwives, counselors, medical specialists in cases of illness, other breastfeeding mothers, and professional lactation consultants. Mothers are also encouraged to seek support from non-governmental organizations, such as La Leche League, which may offer additional guidance and assistance.

The analysed texts depict the mother as someone in need of behavioral prescriptions and continuous expert support, while also being subject to external environmental and social variables, such as access to high-quality childbirth services. Implicit in these recommendations is the assumption that mothers possess the time and financial resources required to care for their own bodies and minds, as well as for their children.

The findings appear to support the argument that the epigenetic paradigm places responsibility for health on the individual, while neglecting its collective and structural dimensions (Meloni, 2014; Meloni and Müller, 2018). At the same time, the active role assigned to mothers in shaping their children’s future health may be understood as a form of “disempowering” empowerment, in which so-called “good” behaviors are mediated and defined by external expert knowledge. Embodied knowledge (Shilling, 2001) - understood as the innate and learned ability to use one’s own body, derived from observing other breastfeeding relationships, maternal instinct, intergenerational transmission of knowledge, and the empathic relationship formed with the child (Stearns, 2013) - stands in contrast to a relationship based on the rational monitoring of physiological signs, such as dehydration or urine output.

“A good indicator of adequate milk intake is the number of wet diapers. on average, you should see 4 or more wet disposable diapers per day, or 6 or more if using cloth diapers”.

It is also important to consider that, although scientific communication aimed at explaining the functions of milk components and nutrients helps make bodily processes more intelligible, it can become complex and difficult for individual mothers without specialized knowledge to critically question.

In sum, the mother is positioned as both a conduit for potential benefits and a source of potential risks to the child, as well as a recipient of external interventions. She is embedded within a complex system in which her role primarily involves the instrumental care of her body, self-surveillance, and reliance on experts who hold implicit authority on the subject.

Within the family environment, the father plays a supporting but significant role. He is encouraged to participate actively in caring for the child through tasks that do not necessarily involve the mother, such as bathing, cradling, and putting the child to sleep. Additionally, he is expected to provide both practical and emotional support to the mother, ensuring she stays hydrated, assisting her physically when she is breastfeeding, and offering encouragement in moments of doubt or discouragement.

He is also assigned the role of protector of the home environment, which is described as a potential source of stress for the mother and a contributor to unhealthy lifestyles. In this vein, he is encouraged to “do the laundry” and “manage relationships with family members”.

“Make sure that the diet and the home environment are healthy. Always keep fresh foods available (like fruits and vegetables), and protect yourself, mom, and the baby from smoke or other harmful substances”.

Finally, he is also encouraged to take care of himself to be able to be a good parent: “It’s important that you take care of yourself by eating well, staying active, and getting rest when you can (and make sure Mom does too). That way, you’ll be able to be the dad you want to be”.

Finally, it is interesting to note that the child is often portrayed as the recipient of benefits rather than as an active participant in the mother–child relationship’s success. At most, the child is portrayed as a developing subject and as a producer of bodily signals that the mother is expected to learn to recognize.

“Responding to your baby’s needs by offering the breast on demand is the best strategy: when hungry, the baby will naturally regulate both the frequency and duration of each feeding. It may take a bit of patience at first, but it’s definitely worth it!”

This finding supports concerns raised by several scholars regarding the effects of medical discourse in positioning children as passive targets of public health interventions across a range of perceived risks (Brady et al., 2015; Lowe et al., 2015; Pentecost and Ross, 2019).

## 5 Conclusion

This article has shown that the FTD discourse reinforces the recommendation widely endorsed by the international medical community (WHO and UNICEF, 2021) to exclusively breastfeed for the first 6 months of life, and to continue breastfeeding up to 2 years of age or until mother and child decide to stop. In doing so, breastfeeding - and, more broadly, feeding with the mother’s milk - is framed as the optimal nutritional practice to protect children from numerous health risks and to support their overall development. Anchored in biomedical and epigenetic paradigms, this discourse establishes a strong causal link between maternal self-care and well-being, the quality of breastfeeding, and the child’s long-term health outcomes. However, the analysis also reveals that maternal health - understood as instrumental to the child’s well-being - is framed primarily as a matter of individual responsibility. At the same time, this narrative tends to overlook the broader social conditions and material constraints that may limit families’ ability to achieve the ideal of maximizing future generational health.

It has also emerged that the FTD discourse conveys two forms of determinism - social and biological- which are often conceptualized as opposites, yet here appear as mutually reinforcing. Social determinism is asserted by establishing a causal link between parenting practices and human development (Brady et al., 2015; Lowe et al., 2015). This logic underpins the expectation that mothers should minimize bodily and environmental risks to optimize their child’s future health. At the same time, while epigenetics promises a more nuanced understanding of health by integrating biological and environmental factors, it also reconfigures traditional biological determinism. Within this framework, the mother is portrayed as both an organic and sentient vector, subject to regulation and discipline in the name of securing the health of future generations (Richardson, 2021). She is thus held responsible for providing a biologically optimal environment capable of “programming” the child’s genome in ways that may mitigate the intergenerational transmission of disease. In this context, breastfeeding is promoted as a technique of health optimisation, and the multidimensional nature of childcare is reduced to its biomedical function. As a result, parenting practices become increasingly medicalised and reframed as public health interventions (Pentecost and Ross, 2019).

What is the social context surrounding the recipients of this narrative? Do women in Italy truly have the necessary resources and support to follow the FTD recommendations, thereby maximizing their ability to positively influence child development and long-term health through breastfeeding? Achieving desirable goals of public health can be challenging due to social constraints, such as short or poorly paid parental leaves, fixed-term jobs, and a lack of policies that enable contemporary women to fulfil their societal roles without sacrificing the beauty of childrearing (Smyth, 2020). In Italy, the social constraints on parenting have a significant role to play in explaining the context of the fertility crisis as they contribute to the pervasive fatigue experienced by working mothers who are referred to as “funanbles” by Save the Children (2025): these mothers endeavour to care for their children with the support of their partners, whose limited participation is gradually increasing; they are required to maintain a daily balance between childcare options (which may have long waiting lists, limited opening hours and significant expenses), uncertain job opportunities, unstable housing, gender disparity in career paths and limited family assistance (e.g., grandparents may still be working and/or living in distant cities). It is interesting to notice that while Italy has adopted since 2007 a national policy for the protection and promotion of breastfeeding, to date it lacks an implementation strategy and dedicated financial resources (IBFAN Asia and IBFAN Italia, 2023). In light of this context, it is understandable that breastfeeding has a suboptimal incidence in Italy, and that there appears to be a correlation between a woman’s decision to breastfeed, her higher social status and level of education (Landini et al., 2019).

In conclusion, one may suggest that the FTD discourse contributes to pushing contemporary mothers into structural tensions between ideal goals and available means, a situation well known in sociology as anomie (Merton, 1938, 1976). One may also suggest that social structures and policies misaligned with the biological timing of reproduction, the physical and psychological demands of childrearing, and its moral or medical prescriptions, contribute to the perception of motherhood as an obstacle to women’s emancipation and engagement in social roles beyond childrearing (Corradi, 2020; Hausman, 2004).

The FTD narrative may inadvertently cause aspiring and new mothers to feel overwhelmed by the social pressures of intensive mothering and healthism permeating all aspects of their personal and family lives. This, in turn, could exacerbate the already widespread perception that childrearing is a burden, an enormous and demanding responsibility, often accompanied by limited support and an overload of recommendations.

## Data Availability

The raw data supporting the conclusions of this article will be made available by the authors, without undue reservation.

## Footnotes

^1^ https://www.statoregioni.it/media/2467/p-3-csr-rep-n-15-20feb2020.pdf?utm_source=chatgpt.com

^2^ https://img.ospedalebambinogesu.it/images/2020/11/02/1824400_1604311048957.pdf?utm_source=chatgpt.com

^3^ https://www.epicentro.iss.it/materno/pdf/parent-opuscolo-allattamento-ottobre-2021-italiano.pdf?utm_source=chatgpt.com

^4^ https://www.savethechildren.it/cosa-facciamo/pubblicazioni/13-atlante-dell-infanzia-a-rischio-come-stai?utm_source=chatgpt.com

^5^ https://millegiorni.info/?utm_source=chatgpt.com

^6^ https://www.epicentro.iss.it/materno/pdf/IN%20PUNTA%20DI%20PIEDI_2025_versione%20digitale.pdf?utm_source=chatgpt.com

^7^ https://www.corriere.it/salute/figli-genitori/guide/24_luglio_18/i-primi-mille-giorni-che-decidono-la-salute.shtml?utm_source=chatgpt.com
